# Bilateral Ureteral Stenosis with Hydronephrosis as First Manifestation of Granulomatosis with Polyangiitis (Wegener's Granulomatosis): A Case Report and Review of the Literature

**DOI:** 10.1155/2020/7189497

**Published:** 2020-12-21

**Authors:** Joelle Suillot, Jürg Bollmann, Samuel Rotman, Eric Descombes

**Affiliations:** ^1^Service of Nephrology, HFR Cantonal Hospital Fribourg, Fribourg, Switzerland; ^2^University of Fribourg, Fribourg, Switzerland; ^3^Service of Urology, HFR Cantonal Hospital Fribourg, Fribourg, Switzerland; ^4^Service of Clinical Pathology, Lausanne University Hospital, University of Lausanne, Lausanne, Switzerland

## Abstract

Ureteral stenosis is a rare manifestation of granulomatosis with polyangiitis (formerly known as Wegener's granulomatosis). We report the case of a 76-year-old woman with progressive renal failure in which bilateral hydronephrosis due to ureteral stenosis was the first manifestation of the disease. Our patient also had renal involvement with pauci-immune crescentic glomerulonephritis associated with high titers of anti-proteinase 3 c-ANCAs, but no involvement of the upper or lower respiratory tract. The hydronephrosis and renal function rapidly improved under immunosuppressive therapy with high-dose corticosteroids and intravenous pulse cyclophosphamide. We reviewed the literature and found only ten other reported cases of granulomatosis with polyangiitis/Wegener's granulomatosis and intrinsic ureteral stenosis: in two cases, the presenting clinical manifestation was unilateral hydronephrosis and in only two others was the hydronephrosis bilateral, but this complication developed during a relapse of the disease. This case emphasizes the importance of including ANCA-related vasculitis in the differential diagnosis of unusual cases of unilateral or bilateral ureteral stenosis.

## 1. Introduction

Granulomatosis with polyangiitis (GPA) (formerly known as Wegener's granulomatosis) is a necrotizing vasculitis of the small to medium vessels, according to the revised International Chapel Hill Consensus Conference Nomenclature of Vasculitides [1]. Its classic triad consists of necrotizing granulomatous vasculitis of the upper and lower respiratory tract in association with pauci-immune crescentic glomerulonephritis [2]. It is often associated with antineutrophilic cytoplasmic antibodies (ANCA) against proteinase 3 (anti-PR3), but occasionally also against myeloperoxidase (anti-MPO) [2]. GPA is an uncommon disease, with an annual incidence of about 10 cases per million inhabitants, a prevalence of 22–157 cases per million [3] and a peak incidence in the fourth and fifth decades of life [4]. Men and women are affected at a similar frequency. GPA was a fatal disease before the introduction of effective immunosuppressive treatments. The use of high-dose corticosteroids associated with cyclophosphamide has markedly improved survival as well as renal survival of patients with ANCA-associated vasculitis [3].

GPA can affect all organs of the body. Although rare, urogenital manifestation can occur during the course of the disease and may be asymptomatic [5]. Granulomatous inflammation of the prostate, bladder, penis, testes, seminal vesicles, ureters, urethra and epididymis have already been reported.

We are reporting on a case in which bilateral hydronephrosis due to bilateral ureteral stenosis was the first clinical manifestation of GPA. Ureteral stenosis is a rare complication of GPA: only 11 cases (including ours) have been reported in the literature to date and, to our knowledge, our case is the first one in which bilateral hydronephrosis was the presenting manifestation of the disease.

## 2. Case Report

A 76-year-old woman was transferred to our hospital after a 6-month history of progressive renal failure with bilateral hydronephrosis of unclear origin and normal cystoscopy.

At admission the patient complained of fatigue, bilateral leg oedema and a 10 kg weight loss. The patient denied any episode of macroscopic hematuria. Clinical examination found a patient in poor general condition with bilateral pitting edema of the legs. Temperature was 36.5°C, blood pressure 135/70 mmHg. The laboratory findings were as follows: serum creatinine was 601 *μ*mol/l, urea 39.5 mmol/l, total protein 57.5 g/l, serum albumin 28 g/l, sodium 143 mmol/l, potassium 4.8 mmol/l. The sedimentation rate was 81 mm/h, C-reactive protein (CRP) 33 mg/l, hemoglobin 107 g/l, leukocyte count 7.2 G/l and platelets 333 G/l. Urinalysis showed microscopic hematuria with mild leukocyturia. Urine culture was sterile. Creatinine clearance was 4 ml/min. Proteinuria was present at 1.9 g/24 h, without paraproteins.

**Figure 1:**
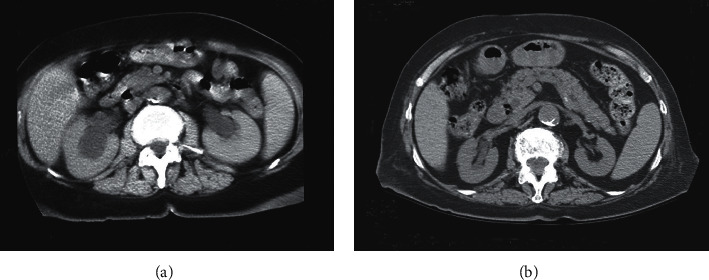
Native CT scan (a) at admission showing massive bilateral hydronephrosis and (b) at 3 months showing a complete regression of the hydronephrosis on the left side and a partial regression on the right side.

**Figure 2:**
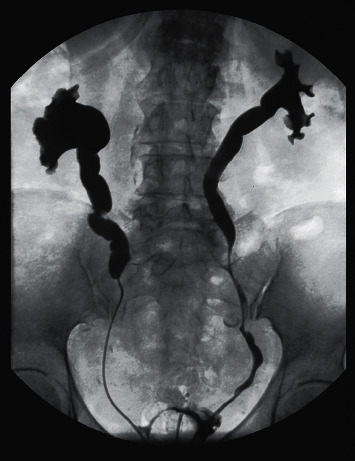
Retrograde pyelography showing bilateral hydro-uretero-nephrosis with a complete ureteral stenosis on the right and a partial ureteral stenosis on the left.

A native abdominal CT scan was performed showing bilateral hydro-uretero-nephrosis with massive dilatation of the upper ureters and of the renal pelvis (Figure 1(a)). The CT scan showed no signs of an obstructive abdomino-pelvic mass, of retroperitoneal fibrosis, urolithiasis or sloughed papillary necrosis. Bilateral ureteral catheterization using double J catheters was performed and the retrograde pyelography revealed that the bilateral hydronephrosis was due to a complete right ureteral stenosis and a partial stenosis on the left side (Figure 2). As bilateral malignancy was considered in the differential diagnosis a small periprocedural ureteral biopsy was performed showing only nonspecific inflammation without tumoral cells.

**Figure 3:**
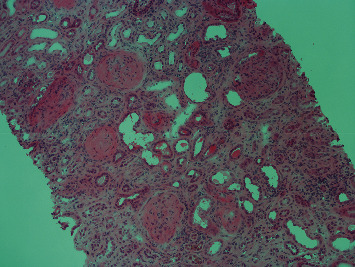
Renal biopsy showing extracapillary crescentic glomerulonephritis involving almost all glomeruli (HE, 100x).

In the following days, despite an improvement of diuresis (≈2000 ml/day) with double J catheters, no improvement of renal function occurred. The diagnostic workup was therefore completed with immunological tests showing high c-ANCA titers (1 : 1280) with anti-PR3 positivity. Thereafter, a kidney biopsy detected pauci-immune crescentic glomerulonephritis (Figure 3). The ENT exam and a native pulmonary CT scan were normal.

Treatment with pulse methylprednisolone (4 × 1 gram) followed by oral prednisone in combination with monthly intravenous pulse cyclophosphamide (3 × 500 mg) was started. Renal function progressively improved in the following weeks and the creatinine stabilized at 140 *μ*mol/l after 3 months, with negative c-ANCA. At the same time repeated ultrasounds showed a progressive regression of the hydro-uretero-nephrosis and the double J catheters were withdrawn. A follow-up CT scan at 3 months showed a complete regression of the hydronephrosis on the left side and a partial regression on the right side (Figure 1(b)). Of note this second CT scan, as the first one, showed no evidence of an obstructive abdomino-pelvic mass, of retroperitoneal fibrosis, urolithiasis or papillary necrosis.

## 3. Discussion

In adults, the most frequent causes of bilateral ureteral obstruction are retroperitoneal or pelvic neoplasms, calculi, or retroperitoneal fibrosis. However, the differential diagnosis also includes other rare etiologies.  Table 1 summarizes the causes of bilateral, or potentially bilateral, ureteral obstruction according to the mechanism of obstruction and shows that different inflammatory or systemic diseases, including small and medium vessel vasculitis, can cause bilateral hydro-uretero-nephrosis. As we will discuss further below, sometimes, unilateral or bilateral ureteral stenosis with hydronephrosis may be due to ANCA-associated vasculitis. To our knowledge, we are reporting on the first patient with bilateral hydronephrosis as the presenting manifestation of ANCA-associated vasculitis.

Urogenital involvement is rare in GPA. Large series of GPA patients have reported between 1% and 10% of cases with urogenital involvement [6, 24]. Cases of asymptomatic urogenital involvement have also been reported in autopsy studies of patients with GPA, and urological manifestation may be underestimated because complete urological investigations are not routinely performed [5]. The main locations of the urogenital manifestations of GPA are summarized in Table 2. Prostatitis is the most common, followed by bladder involvement, orchitis, and penile ulcerations [5]. When urological involvement is present, it is usually observed as part of a generalized systemic disease associated with upper respiratory involvement in 90–100% of the cases, pulmonary lesions in 80%, and glomerulonephritis in 45–60% [5]. Isolated urological manifestations can precede GPA diagnosis in 12–18% of the cases having urological involvement [6, 24]. Intrinsic ureteral stenosis is a rare manifestation of GPA as only 11 cases (including ours) have been reported in the literature to date [6, 24–30]. Huong et al. [6] and Kamar et al. [27] each described two cases. One case was reported twice by the same author [6, 31]. Two other cases were published twice [26, 29, 32, 33] and another case three times [25, 34, 35]. It should be noted that cases of patients with GPA in which hydronephrosis was due to extrinsic ureteral obstruction caused by retroperitoneal inflammation, pseudotumors, or vascular compression as well as prostatic obstruction as a consequence of GPA-related inflammation have also been reported [36–44].

Table 3 summarizes the main clinical features of the 11 cases of ureteral stenosis reported in the literature. In most cases (8/11 = 73%), the ureteral involvement occurred during a relapse of GPA and in only two cases was the involvement bilateral [6, 26]. In the two remaining cases, the ureteral stenosis was, as in our patient, the initial manifestation of GPA, but in these two patients, the hydronephrosis was unilateral [27, 30]. Davenport et al. briefly described a case of initial manifestation of GPA with bilateral ureteral obstruction, but this was due to necrotic debris in the bladder and in the two ureters [43]. Interestingly, a case of ureteral stenosis has also been reported in a kidney transplant due to the recurrence of GPA after transplantation [28].

In 6 of these 11 cases, open ureteral surgery was performed as part of the diagnostic and/or therapeutic workup and granulomatous/vasculitic inflammation was found in all [24, 27–30]. In the other four cases that underwent only an endoscopic workup, no ureteral biopsy was performed [6, 25, 26]. Hence, our case is in fact the only one where a small endoscopic biopsy was performed in order to rule out malignancy. This biopsy showed only nonspecific inflammation, and we consider that our biopsy was too small and superficial to allow the detection of a granulomatous or vasculitic inflammation. However, one may question if the bilateral ureteric stenosis in our patient may eventually be unrelated to the ANCA-associated vasculitis. In this respect, we consider that the accurate initial diagnostic workup, the follow-up exams performed at 3 months, and the favourable response with the regression of the hydro-uretero-nephrosis under immunosuppressive therapy allow to reasonably rule out the alternative diagnosis listed in Table 1.

Concerning ANCAs, ANCA determinations were not performed on the oldest cases reported in the literature as this diagnostic test was not yet available at the time. Except for the two cases reported by Kamar et al., ANCA was positive in all other cases, with anti-PR3 positivity in three patients and anti-MPO positivity in one. The two ANCA-negative cases reported by Kamar et al. are unique since these two patients had isolated 2 and 3 cm long unilateral ureteral stenoses with granulomatous inflammation at surgical resection as the sole manifestation of the disease [27].

The treatment of GPA with a combination of glucocorticoids and immunosuppressants, mainly cyclophosphamide, has been well established. All but three of the reported patients received this treatment and responded well to it (Table 3). To the best of our knowledge, the recurrence of ureteral stenosis upon medical treatment has never been reported. Endoscopic placement of double J catheters was performed in our case, as well as in five others [6, 24, 26], followed by open surgery in two cases [27]. Open surgery was initially performed in four cases [28–30, 35]. The last case underwent no urological therapy as the patient responded rapidly to immunosuppression [6]. In general, surgical repair of the ureters is unnecessary because urological symptoms improve quickly on medical treatment. Consequently, all authors advocate that surgery should only be considered in patients who do not rapidly and effectively respond to the combination of corticoids and immunosuppressants.

In summary, the present review indicates that unilateral or bilateral ureteral stenosis can be the first clinical manifestation of GPA or may occur during a relapse of the disease. Since the clinical presentation may mimic cancer, the right diagnosis can avoid unnecessarily invasive procedures. Therefore, ANCA-related vasculitis should be considered in the differential diagnosis of unusual cases of unilateral or bilateral ureteral stenosis and ANCA testing should be performed in patients in whom a diagnosis is not readily apparent. The data from the reviewed literature suggest that ureteral stenosis responds well and rapidly to glucocorticoids and immunosuppressants.

## Figures and Tables

**Table 1:** Causes of bilateral or potentially bilateral ureteral obstruction^*∗*^.

Urinary tract malformations	Several mainly congenital pediatric diseases
Urolithiasis and endoluminal obstruction	Calculi, papillary necrosis with sloughed papilla, blood clots, fungus balls
Intrinsic ureteral obstruction	Transitional cell carcinoma and other malignant neoplasms fibroepithelial polyps, ureteritis cystica
Extrinsic ureteral obstuction	Abdominopelvic tumors, lymphoma, retroperitoneal fibrosis endometriosis, sarcoidosis
Systemic and inflammatory diseases^*∗∗*^	Small-vessel vasculitis, periarteritis nodosa, Churg–Strauss, Henoch–Schönlein purpura, eosinophilic ureteritis, RA
Ureteral localization of infections	Fungal (actinomycosis), tuberculosis, bacterial, viral (immunocompromised host)
Miscellaneous	Pregnancy, slowed peristalsis, obstructed stent, postoperative

^*∗*^Adapted from references [6–23]. ^*∗∗*^Some can cause either intrinsic or extrinsic obstruction. RA = rheumatoid arthritis.

**Table 2:** Relative frequency of the different urogenital manifestations of granulomatosis with polyangiitis.

Site	Clinical manifestations	Frequency
Prostate	Prostatitis, urinary retention, asymptomatic	+++++
Bladder	Cystitis, pseudotumor	++++
Penis	Ulcerations	+++
Testicles	Orchitis	+++
Kidneys	Pseudotumors, asymptomatic	+++
Urethra	Urethritis	++
Ureter	Stenosis, hydronephrosis	++
Epididymis	Epididymitis	+

Data adapted from Alba et al. [5].

**Table 3:** Summary and characteristics of the reported cases of ureteral stenosis in GPA.

Reference	Year	Age	Sex	Ureteral stenosis		ANCA	Type	Treatment		Course
				Timing	Details			Urological	Medical	
27	2003	59	F	**Inaugural**	Left hydronephrosis with a 3 cm-long iliac ureteral stenosis	Negative		Double J stent, resection of the stenosis by open surgery	No	Remission at 12 months
30	2011	71	F	**Inaugural**	Right hydronephrosis related to a ureteral stenosis	Positive	PR3	Surgical exploration and resection of the stenosis	PRED + CYC	Remission at 6 months
**Our case**		76	F	**Inaugural**	**Bilateral** hydronephrosis related to ureteral stenosis	Positive	PR3	Bilateral ureteral double J stents	PRED + CYC	Remission at 3 months
6, 31^*∗*^	1988	69	F	Relapse	**Bilateral** hydronephrosis related to pelvic bilateral ureteral stenosis	NA		Bilateral ureteral double J stents	PRED + CYC, and plasma exchanges	Died 3 yr later; no urologic relapse
26, 32^*∗*^	2006	38	F	Relapse	**Bilateral** dilatation of both collecting systems with bilateral ureteral stenosis	Positive	MPO	Endoscopic dilatation and double J stent on the left side	PRED + CYC	Remission at 12 months
25, 34^*∗*^, 35^*∗*^	1977	60	F	Relapse	Left ureteral obstruction at the pelvic brim	NA		Transureter ureterostomy	PRED + CYC	Remission at 16 months
29, 33^*∗*^	1982	50	M	Relapse	Moderate dilatation on the right urinary tract	NA		Ureteral resection	PRED + CYC	Remission at 10 yr
28	1994	25	F	Relapse	Hydronephrosis in a transplanted kidney by ureteral obstruction at the ureterovesical junction	Positive	NA	Ureteral resection	PRED + CYC + AZA	Remission at 15 yr
6	1995	55	M	Relapse	Right-sided ureteral stenosis	Positive	NA	None	PRED	Remission at 5 yr
27	2003	21	M	Relapse	Isolated right hydronephrosis with a 2 cm-long stenosis of the iliac ureter	Negative		Double J stent followed by open surgery excision	PRED + CYC	Remission at 6 months
24	2012	53	M	Relapse	Right-sided hydronephrosis with ureteral stenosis	Positive	PR3	Ureteral catheter	PRED and MTX	Died 3 yr later; no urologic relapse

^*∗*^Cases reported two or three times. F: female, M: male, NA: not available, PRED: prednisone, CYC: cyclophosphamide, MTX: methotrexate, AZA: azathioprine, and yr: year.
